# Clinical Efficacy of 5-Aminolevulinic Acid Photodynamic Therapy in the Treatment of Oral Leukoplakia: A Prospective Study with Long-Term Follow-Up

**DOI:** 10.3290/j.ohpd.c_2827

**Published:** 2026-09-29

**Authors:** Jinjin Zhao, Yang Cai, YongXin Hu, Xun Wang, Hong Lu, Ting Liu

**Affiliations:** a Jinjin Zhao Dentist, Affiliated Stomatological Hospital of Guizhou Medical University, Guizhou Medical University, Guiyang, China. Study idea and concept, data analysis and interpretation, drafted the manuscript.; b Yang Cai Professor, Affiliated Stomatological Hospital of Guizhou Medical University, Guizhou Medical University, Guiyang, China. Data analysis and interpretation.; c YongXin Hu Dentist, Department of General-Dentistry II, Affiliated Stomatology Hospital of Guangzhou Medical University, Guangdong Engineering Research Center of Oral Restoration and Reconstruction, Guangzhou Key Laboratory of Basic and Applied Research of Oral Regenerative Medicine, Guangzhou, Guangdong, China. Designed the study, performed the research, analyzed the data.; d Xun Wang Dentist, Affiliated Stomatological Hospital of Guizhou Medical University, Guizhou Medical University, Guiyang, China. Performed the research, analyzed the data.; e Hong Lu Dentist, Affiliated Stomatological Hospital of Guizhou Medical University, Guizhou Medical University, Guiyang, China. Conceptualization, methodology, resources.; f Ting Liu Dentist, Affiliated Stomatological Hospital of Guizhou Medical University, Guizhou Medical University, Guiyang, China. Reviewed and edited the manuscript, read and agreed to the published version of the manuscript.

**Keywords:** 5-aminolevulinic acid, oral leukoplakia, photodynamic, therapy.

## Abstract

**Purpose:**

To observe the clinical efficacy and influencing factors of 5-aminolevulinic acid photodynamic therapy (5-ALA-PDT) in treatment oral leukoplakia (OL), so as to provide therapeutic references for clinical application.

**Materials and Methods:**

After optimizing the 5-ALA-PDT treatment process, 31 OL patients were locally treated with 5- ALA-PDT (wavelength, 635 nm; energy density, 100 J/cm^2^), with 3 sessions per course of treatment. Efficacy was evaluated one month post-treatment by assessing lesion changes. Additionally, the factors that might influence therapeutic efficacy were analyzed.

**Results:**

An overall response rate of 90.3 % was achieved in this study, including 19 patients with complete remission, 9 with partial remission, and 3 with stable disease.The patients were followed for a median of 15 months (95% CI: 9–20 months; range: 3–25 months), during which recurrence occurred in 3 patients and malignant transformation in 2 patients. The treatment effect appeared better for lesions on the ventral tongue than at other sites. Patients who experienced local symptoms such as pain or ulcers after 5-ALA-PDT showed a more favorable response than those without any postoperative reactions. Patients with heterogeneous oral leukoplakia and moderate epithelial dysplasia had a higher recurrence rate than those with homogeneous lesions, simple hyperplasia, or mild epithelial dysplasia, with the peak of recurrence registered at 1–6 months after treatment.

**Conclusion:**

5-ALA-PDT appears to be a promising treatment for OL. However, recurrence and malignant transformation remain concerns.

Oral leukoplakia (OL) is a common precancerous lesion of the oral cavity. It is characterized by white patches on the oral mucosa that cannot be wiped away and cannot be clinically or pathologically diagnosed as any other disease.^3^ The malignancy rate of OL is approximately 7.5%.^21^ Traditional treatments for OL include medication and surgery. However, pharmacological interventions often yield uncertain efficacy and are associated with adverse reactions. Surgical treatment may affect esthetics and lead to functional disorders, such as limited mouth opening and poor articulation.^4,22^ Therefore, identifying an effective and safe therapeutic technique for OL remains an urgent clinical challenge.

Photodynamic therapy (PDT) is a novel treatment approach that combines special photosensitive substances with specific wavelength lasers to produce photodynamic reactions. This approach selectively targets hyperproliferative cells or tissues, while causing minimal damage to normal tissues.^11,13^ 5-Aminolevulinic acid (5-ALA) is a second-generation photosensitizer and a precursor drug that does not have photodynamic activity on its own. It transforms into the endogenous photosensitive substance Protoporphyrin IX (Pp IX)^7,26^ under the action of a series of heme-synthesis enzymes both within and outside the mitochondria. When PpIX is irradiated with light of a specific wavelength, it emits red fluorescence, a property that enables the identification of PpIX-accumulating tissues. Photodynamic fluorescence diagnosis is fundamentally based on this specific accumulation of photosensitive PpIX.^16^ As the most commonly used photosensitizer for PDT treatment of OL, 5-ALA offers several advantages, including a short photosensitivity period, good tissue penetration, high singlet oxygen production, strong targeting ability, and rapid clearance from cells and tissues.^26^

Numerous studies have demonstrated the high feasibility of 5-aminolevulinic acid photodynamic therapy (5-ALA-PDT) for clinical application in treating OL.^5,15,19,23^ However, most of these studies have only observed short-term clinical efficacy, post-treatment recurrence, and malignant transformation. The factors affecting the clinical efficacy of 5-ALA-PDT in treating OL remain unclear. Moreover, moisture isolation and fixation are crucial in the 5-ALA-PDT procedure. Therefore, our study aimed to explore these influencing factors by optimizing the 5-ALA-PDT treatment process and discussing the effectiveness and safety of the treatment, thereby providing new data support for clinical application.

## MATERIALS AND METHODS

### Patients

Based on previous literature, the estimated difference in lesion area before and after treatment was 3.50 ± 5.10 cm^2^. With a two-sided α = 0.05 and a power (1−β) = 0.90, the required sample size was calculated to be 25 cases using the “Test for Paired Means” module in PASS version 15.0.5 (NCSS, LLC; Kaysville, UT, USA). To account for a 20% dropout rate, 32 patients were planned for enrollment.^17^ Of the 34 patients initially assessed, 2 were excluded because of uncontrolled severe systemic disease, and 1 was lost to follow-up. The reason for loss to follow-up was documented and is presented in the CONSORT flow diagram (Fig 1). Thirty-one patients with OL who visited in the Department of Oral Mucosal Diseases of the Affiliated Stomatological Hospital of Guizhou Medical University, Guiyang, Guizhou, China from December 2019 to May 2022 were included in the study. The inclusion criteria were as follows: clinical diagnosis and histopathological examination confirming oral leukoplakia, patients who smoked were required to abstain for at least one month prior to treatment, and age over 18 years. The exclusion criteria included pregnant or breastfeeding women, patients with uncontrolled severe systemic diseases (such as severe liver or kidney damage or malignant tumors), allergies to light, porphyrin, or anesthetic drugs, and those with mental illnesses.

**Fig 1:**
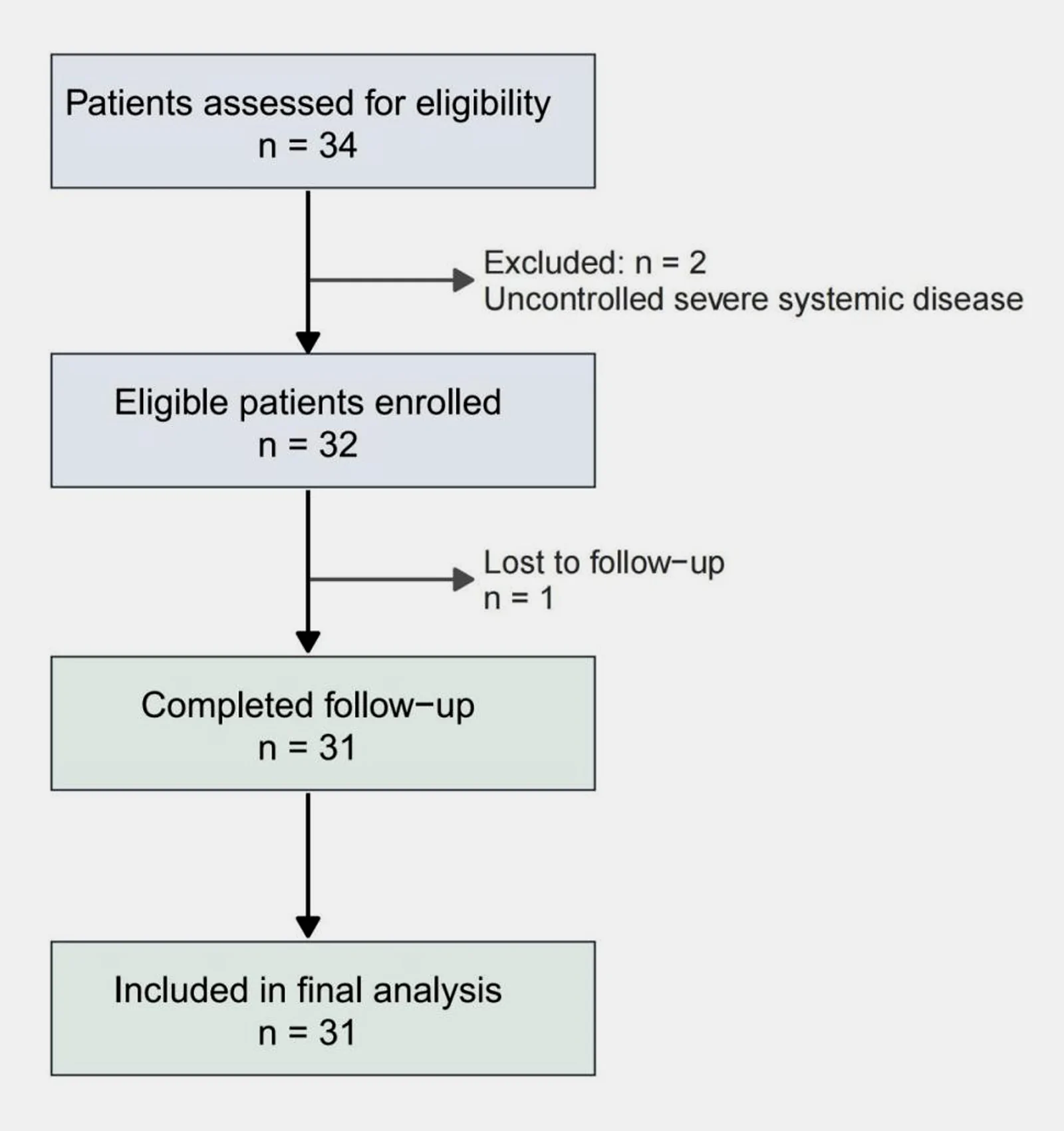
Patient recruitment and follow-up CONSORT flow chart.

This study was approved by the Ethics Committee of the Affiliated Stomatological Hospital of Guizhou Medical University. All patients provided written informed consent for photodynamic therapy.

### Drugs and Equipment

This study employed the following substances and devices: aminolevulinic acid (Fudan Zhangjiang Bio-Pharmaceutical; Shanghai, China), thermosensitive gel (Fudan Zhangjiang Bio-Pharmaceutical), semiconductor laser treatment instrument set at a wavelength of 635 nm±5 nm (Yage Optoelectronic Technology; Wuhan, China), UV purple flashlight with a wavelength of 395 nm (Guangzhongdao Electronics; Shenzhen, China).

### PDT Procedure

Preoperative photographs were taken, and the lesion area was measured using Photoshop software (Adobe; San Jose, CA, USA). 5-ALA powder (118 mg per vial) was dissolved in 0.5 ml of thermosensitive gel to obtain a 20% solution. An impression compound (Shanghai Rongxiang Dental Material; Shanghai, China) was softened in hot water (60° to 70°C).

Sterile cotton soaked with 20% 5-ALA was applied to the oral leukoplakia, fully covering the lesion and extending 3 to 5 mm beyond its margins. The dressing was secured with 4 to 5 layers of glutinous rice paper and then covered with plastic wrap (Fig 2a).

**Figs 2a to 2f:**
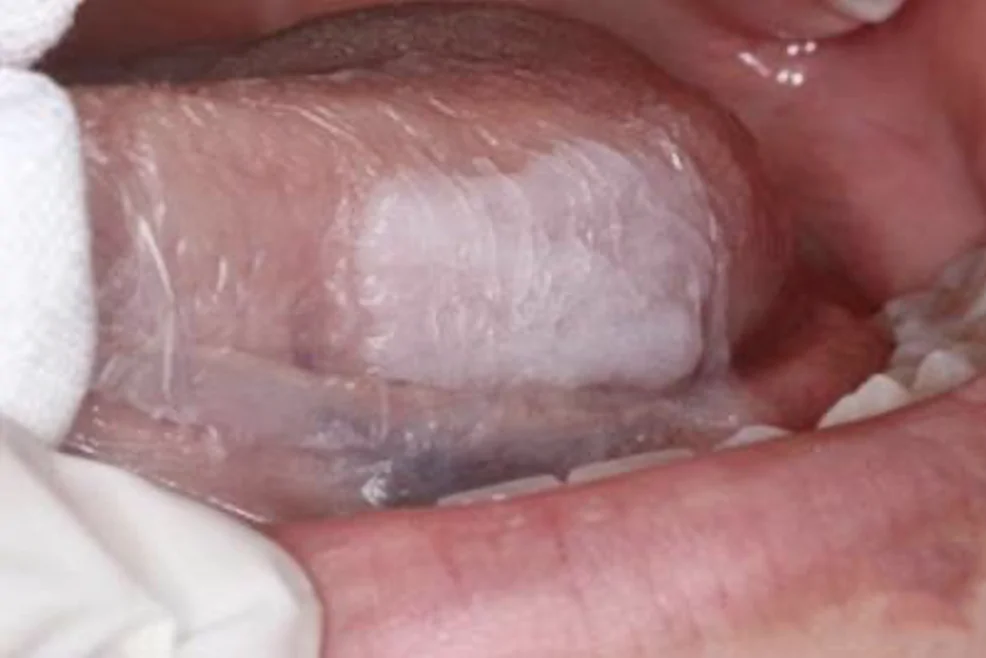

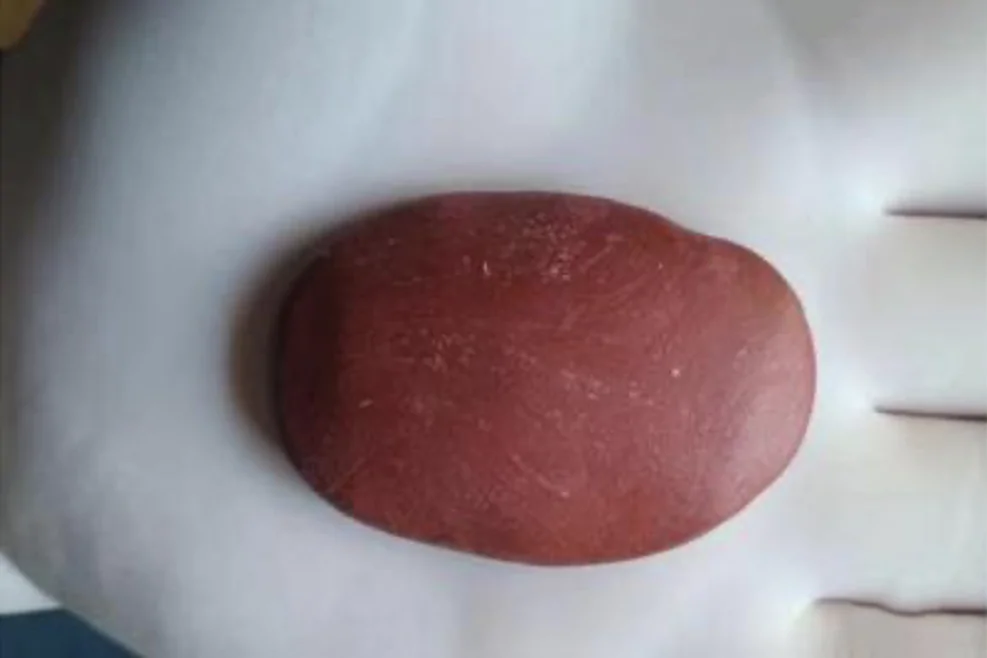

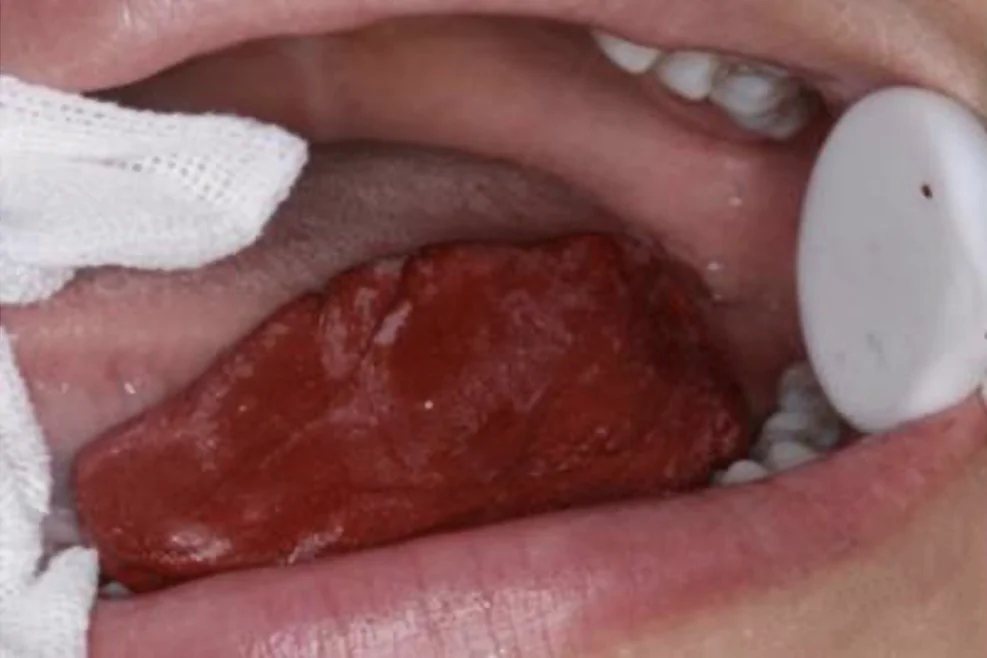

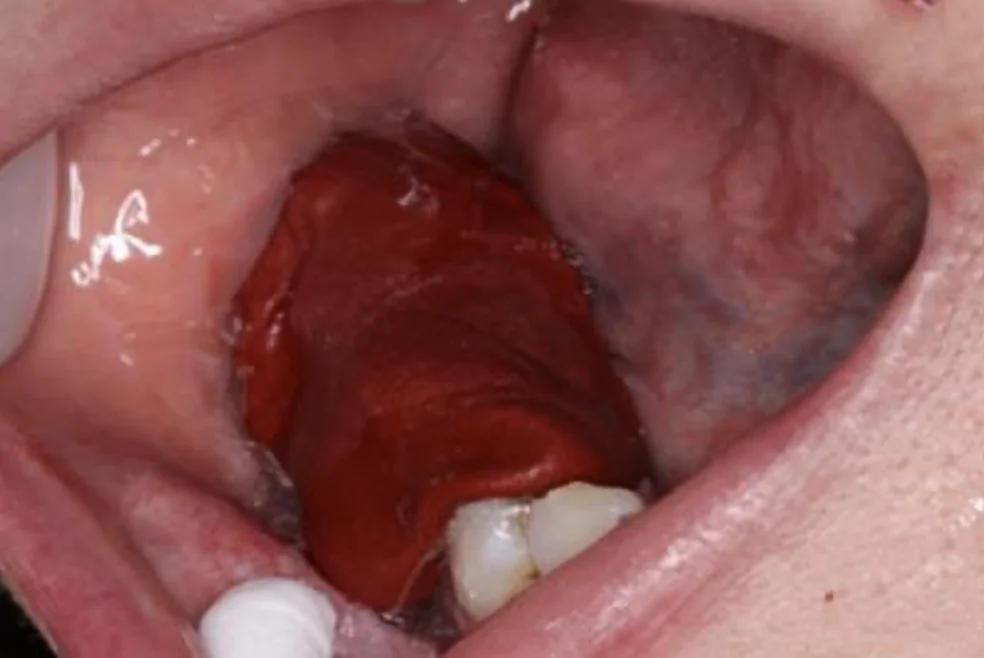

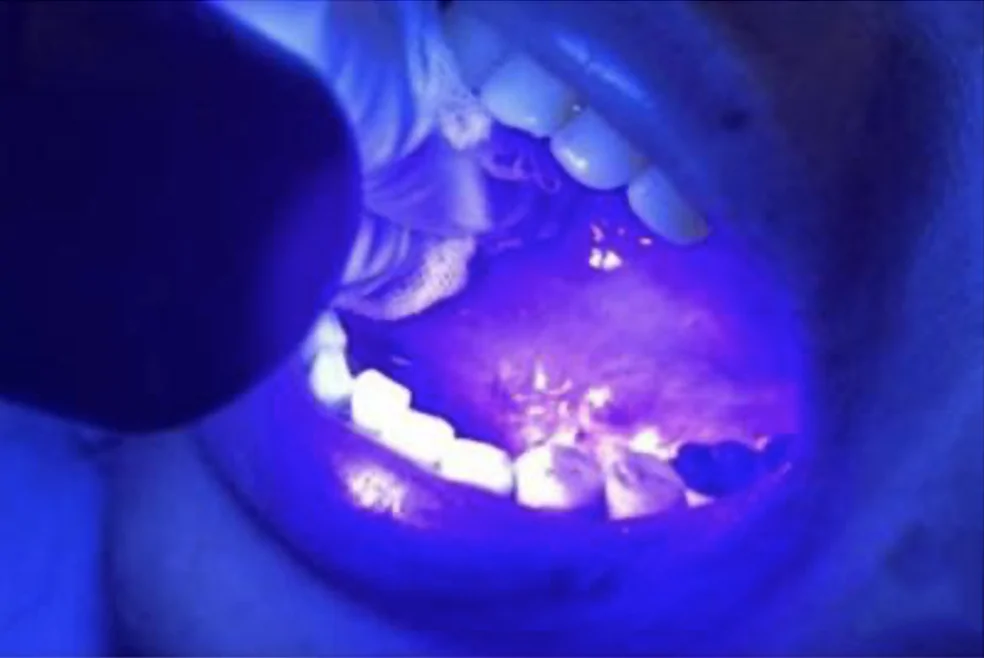

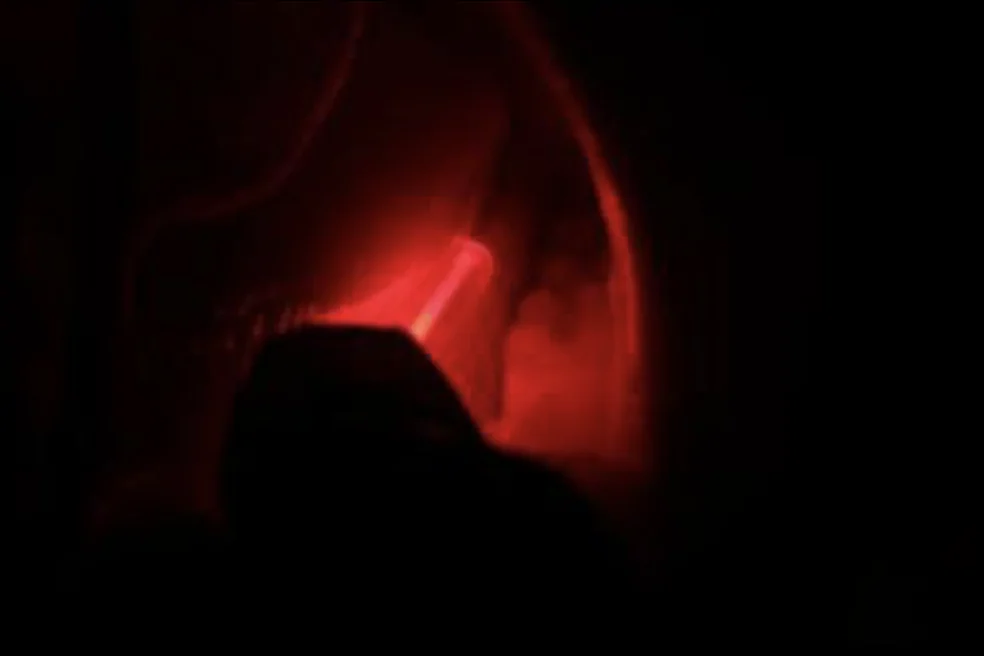
The procedure of administrating topical 5-aminolevulinic acid photodynamic therapy (5-ALA-PDT). a: Moisture isolation and local application of photosensitizer; b: mold impression compound adapted into different shapes according to the lesion’s location; c: the photosensitizer was fixed with red and white proofing paste; d: The photosensitizer was secured with compound within the teeth‑cheek space; e: fluorescence detection; f: the lesion is illuminated with red light for 20 min.

For fixation, the softened impression compound was molded according to the lesion site (Fig 2b). For ventral tongue lesions, an oval shape was made (thickness 5 to 10 mm, width 10 to 15 mm beyond the lesion borders). It was then placed flat over the wrapped dressing, without exceeding the inner border of the teeth. After cooling, the compound hardened, using the gap between the teeth and tongue to maintain stable contact and prevent displacement (Fig 2c). For gingival lesions, an elongated block (same dimensions) was placed over the dressing and extended over the occlusal surfaces. The patient was asked to bite down gently, securing the compound in the space between the teeth and cheek (Fig 2d). To manage salivary contamination, the patient’s head was tilted slightly to the contralateral side, and a saliva ejector was placed at the contralateral mouth angle to remove flowing saliva. The total application time was 3 h; after 1.5 h, the photosensitizer-soaked cotton was replaced, and the fixation process was repeated to ensure consistent tissue contact throughout.

After 3 h, the dressing was removed, and the lesion was irradiated perpendicularly with a UV flashlight. Red fluorescence indicated good photosensitizer absorption (Fig 2e). Conversely, the absence of red fluorescence was considered to indicate poor photosensitizer absorption, and patients in which fluorescence was lacking were excluded.^16^

Under local anesthesia, the lesion was illuminated with a laser (wavelength 635 nm, energy density 100 J/cm^2^) for 20 min. Specifically, the irradiation was carried out in cycles of 5-min illumination, with 3-min intervals between each cycle (Fig 2f).

### Follow-up 

Treatment was conducted every 7 to 10 days, with three sessions constituting one course of treatment. At each follow-up session, the lesion areas were measured. According to the condition of the lesion, each patient underwent 1 or 2 treatment courses. If there were no apparent OL lesions post-treatment, the treatment could be temporarily halted, with efficacy evaluated at the first month post-treatment. If the change in lesion area was less than 30% after three consecutive 5-ALA-PDT treatments, the treatment was discontinued. Patients were re-examined monthly for the first six months post-surgery and every three months thereafter.

### Clinical Efficacy valuation 

Efficacy was assessed one month after the completion of treatment. According to the Expert Consensus on 5-Aminolevulinic Acid Photodynamic Therapy for Oral Potentially Malignant Disorder,^14^ the clinical evaluation criteria for treatment response were as follows: complete remission (CR), defined as complete disappearance of the treated lesion; partial response (PR), defined as at least a 30% reduction in lesion size compared with baseline; stable disease (SD), defined as no change in lesion size or type; and progressive disease (PD), defined as the appearance of new lesions or an increase in lesion area of at least 20% compared with baseline.

### Statistical Analysis 

Statistical analysis was conducted using SPSS software version 25.0 (IBM; Armonk, NY, USA). Measurement data conforming to normal distribution were expressed as mean ± standard deviation, while non-normally distributed measurement data or rank data were described using the median M (Q25, Q75). Countable data were described using frequency and percentage. For within-group comparisons, the paired rank-sum test was used. For measurement data, parametric (independent samples t-test, ANOVA) or non-parametric tests (Mann-Whitney U test, Kruskal-Wallis H-test) were used to compare the number of treatments between different groups. The chi-squared test, corrected chi-squared, and Fisher’s exact test were used to compare differences between groups. A p-value < 0.05 was considered statistically significant.

## RESULTS

### General Information 

A total of 31 patients were included in this study, consisting of 21 males and 10 females. The average age was 51.62±18.41 years. OL occured on the ventral surface of the tongue, the floor of the mouth, cheek, back of the tongue, the gingiva, and the inner corner of the mouth. Among these sites, OL occurred most frequently on the ventral surface of the tongue (17 cases). Pathological examination revealed that 23 patients had simple hyperplasia, 6 had mild epithelial dysplasia, and 2 had moderate epithelial dysplasia. Statistical analysis was conducted on the general data of 31 patients (Table 1).

**Table 1:** Basic clinical characteristics of patients with oral leukoplakia OLs (n=31)

Case characteristics	Number of cases (%)	Case characteristics	Number of cases (%)
**Gender**		Smoking history	
Male	21 (67.74)	Yes	24 (77.42)
Female	10 (32.26)	No	7 (22.58)
**Age (years)**		Drinking history	
<50	15 (48.39)	Yes	23 (74.19)
≥50	16 (51.61)	No	8 (25.81)
**Lesion location**		Chewing betel nut	
Tongue dorsal surface	17 (54.84)	Yes	9 (29.03)
Floor of mouth	2 (6.45)	No	22 (70.97)
Other	12 (38.71)	Lesion size	
**Lesion classification**		<2cm^2^	14 (45.16)
Homogeneous	25 (80.65)	≥2cm^2^	17 (54.84)
Non-homogeneous	6 (19.35)		
**Pathological characteristics**			
Simple hyperplasia	23 (74.19)		
Mild to moderate epithelial dysplasia	8 (25.81)		


### Clinical Efficacy of 5-ALA-PDT 

Before treatment, the range of OL lesions was 0.52 cm^2^ to 5.43 cm^2^, with a median of 2.14 cm^2^. After treatment, the range was 0 cm^2^ to 3.68 cm^2^, with a median of 0.15 cm^2^. The post-treatment lesion area was statistically significantly smaller than the pre-treatment lesion area (p<0.01) (Table 2). Among the 31 responding patients, the treatment effectiveness rate was 90.3%. 61.3% (19/31) of the patients achieved CR, 29.0% (9/31) experienced PR, and 9.7% (3/31) of them experienced SD (Fig 3).

**Table 2:** Comparison of lesion area before and after 5-ALA-PDT treatment

Number of lesions	Preoperative lesion area in cm^2^	Postoperative lesion area in cm^2^	Z	p
31	2.14 (1.55, 2.75)	0.15 (0.00, 0.3)	-4.86	<0.01
Statistically significant difference at p<0.05. Values in parentheses are 25th and 75th percentiles.				

**Figs 3a to 3f:**
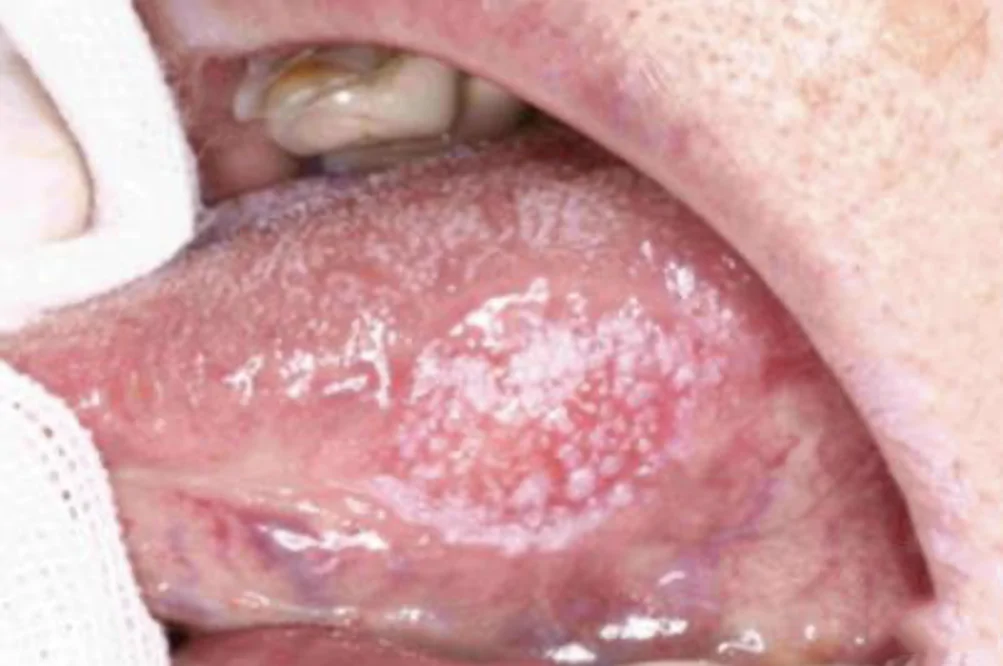

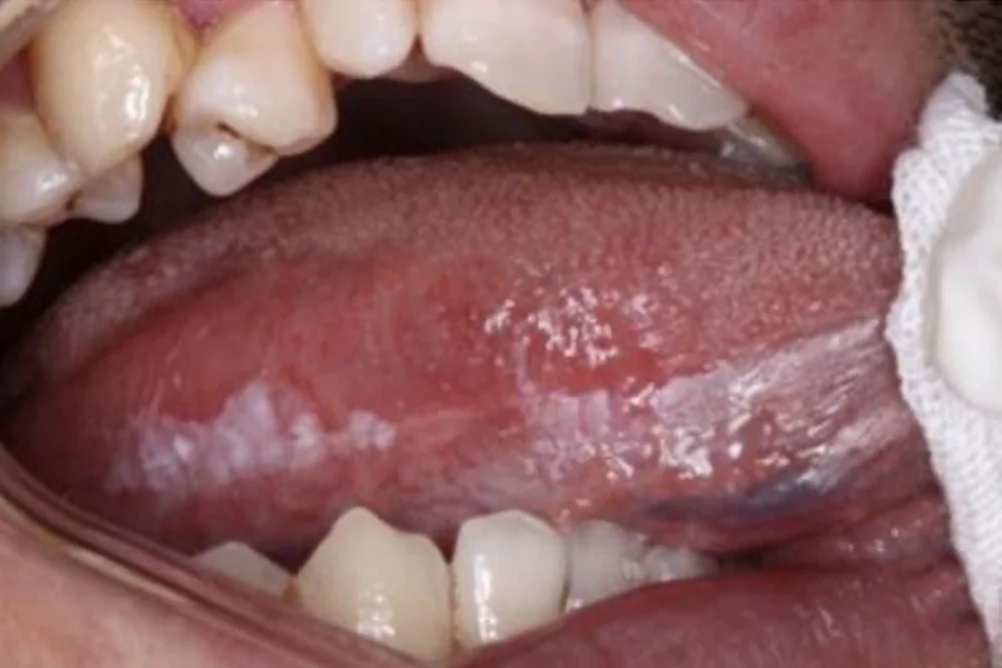

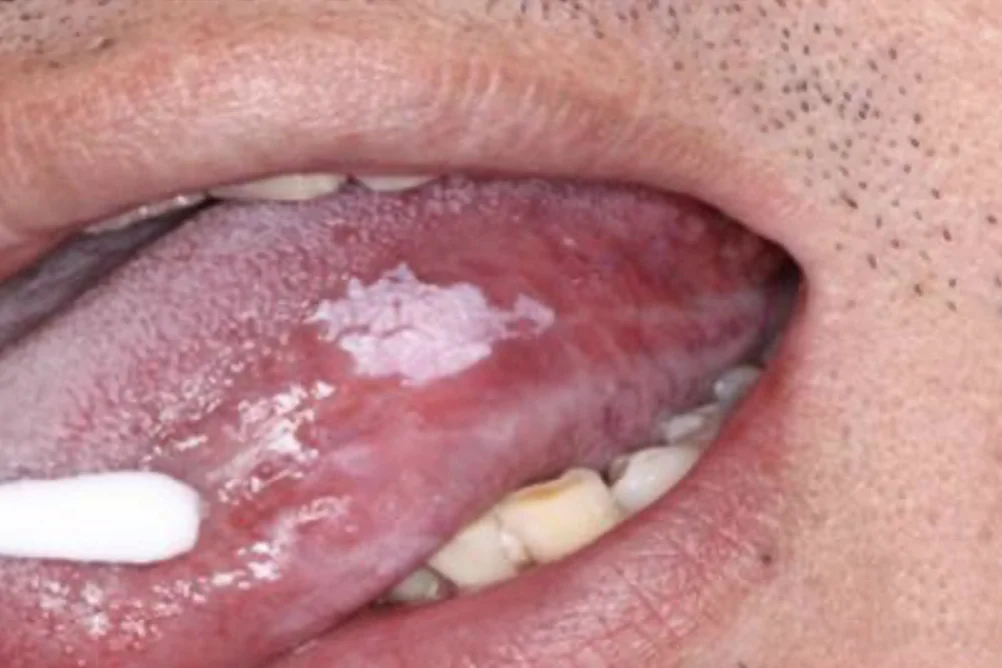

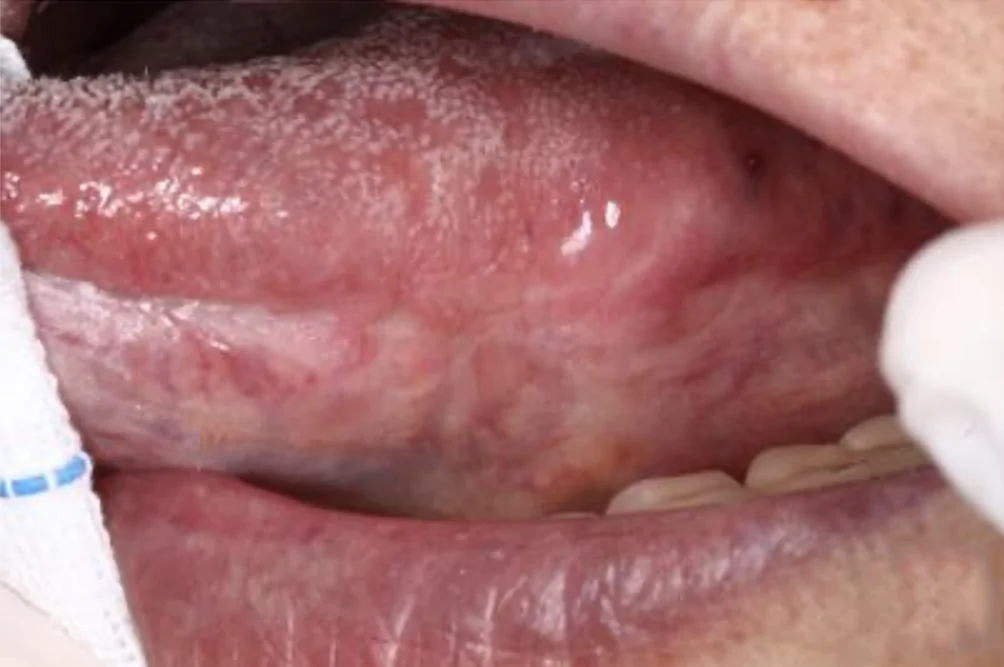

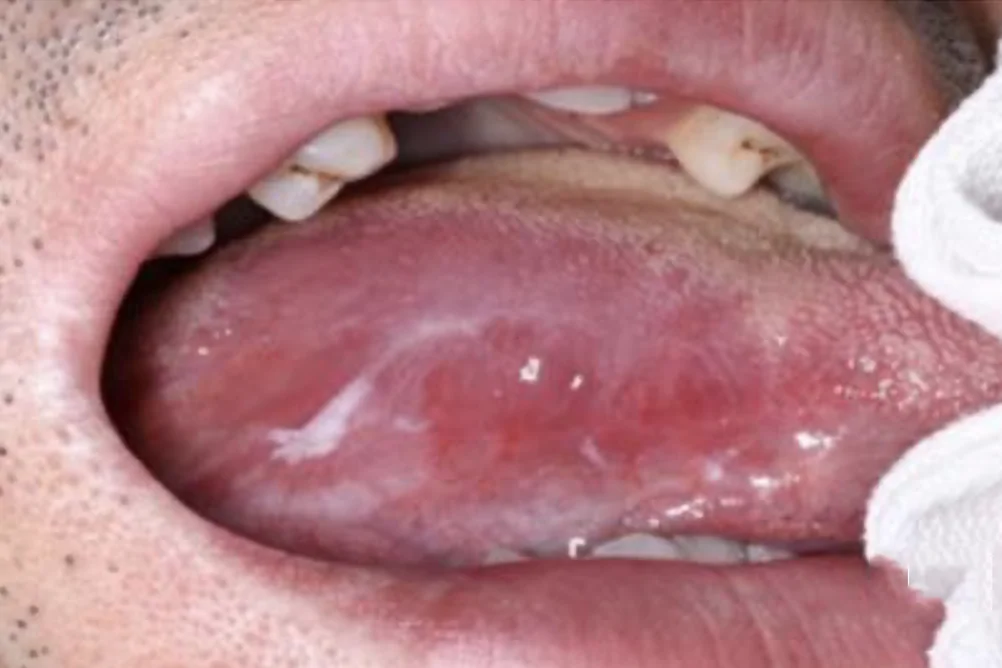

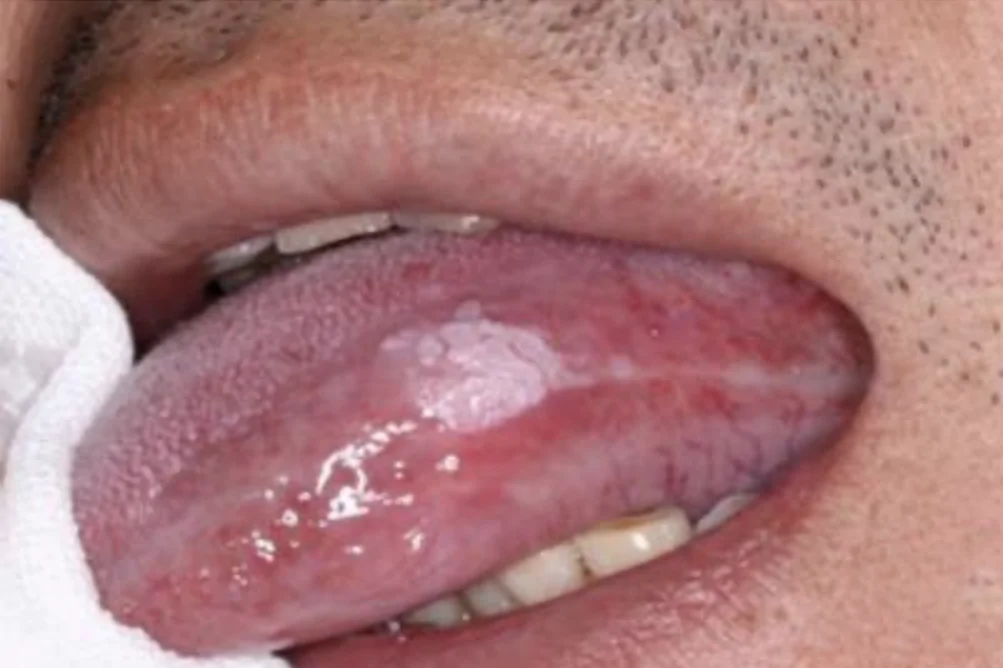
Representative images showing different clinical outcomes. a: leukoplakia on the ventral tongue; b: leukoplakia on the ventral tongue; c: leukoplakia on the dorsum of the tongue; d: the white plaque on the tongue’s ventral mucosa completely subsided; e: the white plaque on the tongue’s ventral mucosa partially subsided; f: no significant change in the white plaque on the dorsum of the tongue after 5-ALA-PDT.

### Analysis of Factors Affecting the Efficacy of 5-ALA-PDT

This analysis demonstrated that although certain factors, such as lesion location and postoperative reactions, statistically significantly influenced the efficacy of 5-ALA-PDT in treating OL, other factors, including gender, degree of abnormal growth, smoking history, lesion type, and lesion size, showed no statistically significant association with treatment outcomes (Table 3).

**Table 3:** The association of OL clinical basic characteristics and 5-ALA-PDT therapeutic effect (n=31)

Basic clinical characteristics		Therapeutic effect			Chi-squared value	p-value
		Cases (n)	Effective cases (n)	Effective invalid cases rate (%)		
Gender	Male	18	3	85.7	-	0.533**
	Female	10	0	100		
Age in years	<50	13	2	86.7	0.003	0.953*
	≥50	15	1	93.8		
Lesion location	Ventral tongue	17	0	100	10.473	0.003^&^**
	Floor of the mouth	0	2	0		
	Other	11	1	91.7		
Pathological characteristics	Simple hyperplasia	22	1	95.7	4.147	0.156**
	Mild epithelial dysplasia	4	2	66.7		
	Moderate epithelial dysplasia	2	0	100		
Lesion classification	Homogeneous	22	3	88	-	1.000**
	Non-homogeneous	6	0	100		
Smoking history	Yes	21	3	87.5	-	1.000**
	No	7	0	100		
Drinking history	Yes	20	3	87	-	0.550**
	No	8	0	100		
Chewing betel nut	Yes	8	1	88.9	-	1.000**
	No	20	2	90.9		
Lesion size	<2 cm^2^	13	1	92.9	0	1.000*
	≥2 cm^2^	15	2	88.2		
Clinical symptoms (side effect)	Yes	28	1	96.6	-	0.006^&^**
	No	0	2	0		
*Expressed using corrected chi-squared; **expressed using Fisher’s exact probability method; ^&^ statistically significant, p<0.05.						

### Assessment of Adverse Reactions 

Among the 31 patients, 25 experienced local pain and ulcers post-operatively. In addition to pain and ulcers, 4 patients also had mild local swelling, whereas 2 patients showed no statistically significant local reactions (Table 4).

**Table 4:** Local clinical symptoms (side effect) after 5-ALA-PDT

Clinical symptoms (side effects)	Cases (n)	Time to evoke a local response	Time for local response to subside
Pain	25	1-3 h after surgery	3-5 days after surgery
Ulcers	25	24-48 h after surgery	7-10 days after surgery
Swelling	4	24-48 h after surgery	3-5 days after surgery
No response	2		


### Analysis of OL Recurrence and Influencing Factors 

Kaplan-Meier survival analysis showed that 3 recurrences among the 31 patients during the follow-up period. All recurrence events were observed within the first 4 months, and the overall recurrence-free survival rate during follow-up was approximately 90% (Fig 4). The comparison of the recurrence of OL lesions with basic clinical characteristics revealed that the recurrence rate was associated with pathological manifestations and clinical features (p<0.05). However, there was no statistically significant difference in the recurrence of OL lesions with respect to lesion location, age, gender, smoking history, lesion size, and post-treatment reactions (p>0.05) (Table 5).

**Fig 4:**
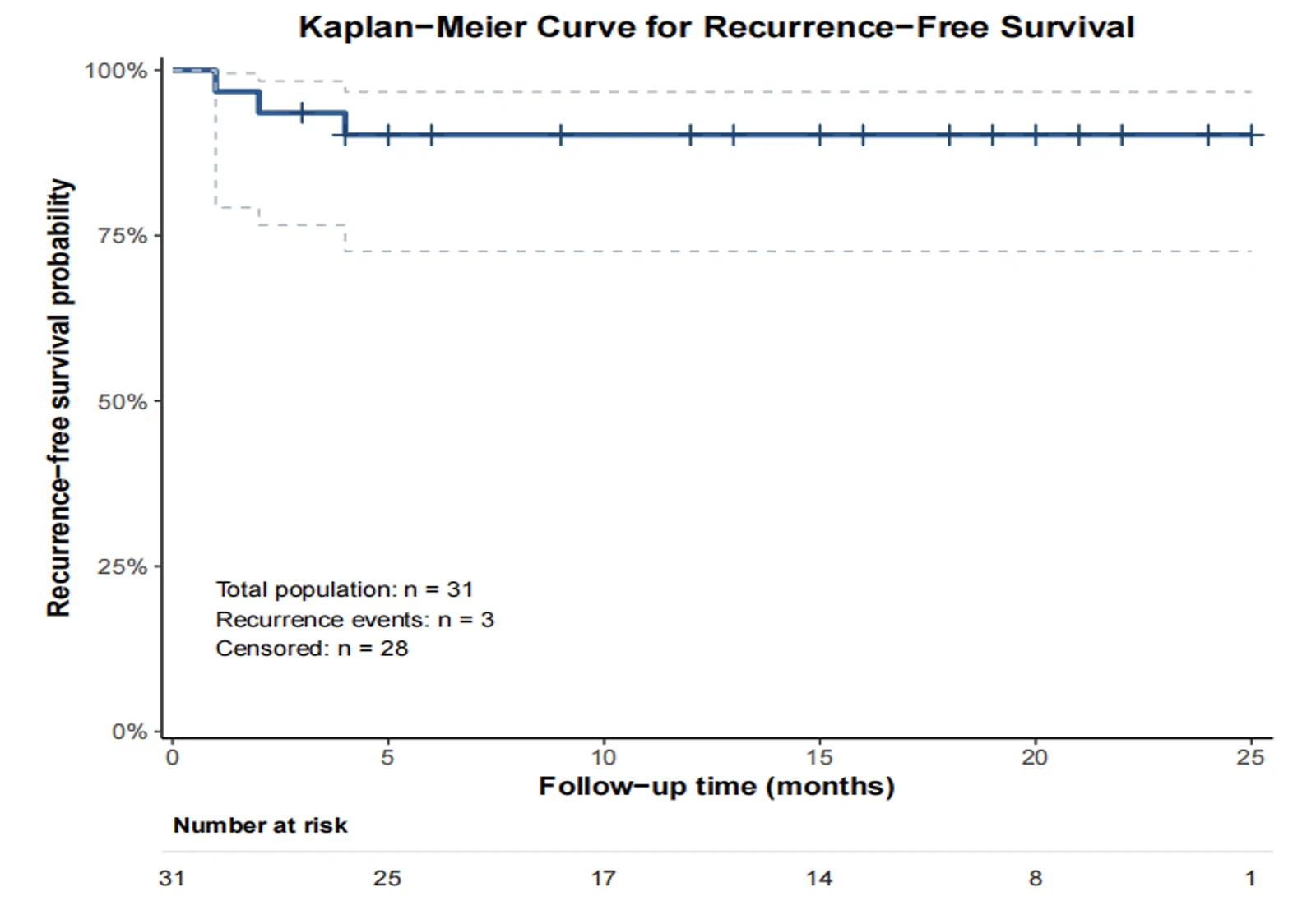
Kaplan-Meier curves for time to recurrence indicated that recurrences primarily occur within the first 4 months.

**Table 5:** The association of clinical basic characteristics and recurrence of patients with OL (n=31)

Basic clinical characteristics		Recurrence			Chi-squared value	
		Yes	No	Recurrence rate (%)		
Gender	Male	2	19	9.5	-	1.000**
	Female	1	9	10		
Age (Year)	<50	1	14	6.7	-	1.000**
	≥50	2	14	12.5		
Lesions location	Ventral tongue	3	14	17.6	2.399	0.387**
	Floor of the mouth	0	2	0		
	Other	0	12	0		
Pathological characteristics	Simple hyperplasia	1	22	4.3	9.563	0.011^&^**
	Mild epithelial dysplasia	0	6	0		
	Moderate epithelial dysplasia	2	0	100		
Lesion classification	Homogeneous	1	24	4	4.763	0.029^&^**
	Non-homogeneous	2	4	33.3		
Smoking history	Yes	2	22	8.3	-	0.550**
	No	1	6	14.3		
Drinking history	Yes	2	21	8.7	-	1.000**
	No	1	7	12.5		
Chewing betel nut	Yes	1	8	11.1	-	1.000**
	No	2	20	9.1		
Lesion size	<2 cm²	0	14	0	1.089	0.297*
	≥2 cm²	3	14	17.6		
Clinical symptoms (side effect)	Yes	3	26	10.3	-	1.000**
	No	0	2	0		
*Corrected chi-squared test; **Fisher’s exact test; ^&^p < 0.05 was considered statistically significant.						

### Malignant Transformation

During follow-up, malignant transformation occurred in two patients (malignant transformation rate: 6.5%), and their clinicopathological parameters are listed in Table 6.

**Table 6:** Clinicopathological parameters of the two patients with malignant transformation

Gender	Age in years	Smoking history	Drinking history	Chewing betel nut	Lesion location	Pathological characteristics	Malignant transformation time (month)
Male	28	Yes	Yes	Yes	Ventral part of the tongue	Simple hyperplasia	18
Male	67	Yes	Yes	No	Ventral part of the tongue	Moderate epithelial dysplasia	14


## DISCUSSION

Although 5-ALA-PDT has been used to treat OL for over a decade, detailed reports on its technical procedures remain scarce. Moreover, variations in treatment protocols and parameters among different studies have led to considerable differences in the specific procedures employed by physicians and, consequently, in clinical treatment outcomes. Firstly, this study utilized photoshop software to measure the lesion area, demonstrating that it is more accurate than traditional periodontal probes in reflecting changes in lesion size, thus facilitating data collection and analysis. Secondly, building upon the 5-ALA-PDT treatment guidelines for OL established by Chen et al,^6^ this study further optimized the treatment process to enhance the clinical efficacy of 5-ALA-PDT for OL. Glutinous rice paper, due to its safety and edibility, has been widely used in previous studies for fixing photosensitizer dressings.^8,18^ Our preliminary research found that initial fixation with glutinous rice paper and gauze was effective.

However, for areas with greater mobility, such as the underside of the tongue or cheeks, the use of only glutinous rice paper and gauze was insufficient for achieving secure fixation. Therefore, this study utilized a combination of glutinous rice paper and impression compound for dressing fixation. Before replacing the 5-ALA impregnated cotton midway, the position of the dressing was checked. It was found that approximately 90% of patients had no dressing displacement. This study is the first to use impression compound as a fixation material in 5-ALA-PDT treatment, mainly due to its excellent adhesive properties. The compound allows saliva to flow easily along its edges, reducing local drug dilution and enhancing patient comfort. The impression compound used is a common dental impression material known for its safety, convenience, and high malleability.

Nevertheless, previous studies did not mention replacing the photosensitizer cotton midway through the process. Our preliminary research found that replacing the 5-ALA cotton midway increases the drug saturation, thereby enhancing the photodynamic effect. During the illumination process, this study adopted an intermittent laser-irradiation method, with a 3-min break every 5 min. This was mainly because this mode might alleviate pain during the irradiation process and allow epithelial cells in the lesion to accumulate new oxygen.^5,8^ This study achieved an effectiveness rate of 90.3%, with a complete remission rate of 61.3% and a partial remission rate of 29.0%. Comparatively, Han et al^8^ treated 29 OL patients with 5-ALA-PDT, achieving a total effectiveness rate of 86.2%, a complete remission rate of 55.2%, and a partial remission rate of 31.0%. The slightly higher effectiveness rate in our study may be due to the optimized treatment process.

Our study also compared the clinical efficacy of OL treatment with postoperative local reactions. We found that patients who had no postoperative reactions showed poor clinical outcomes, while those who developed ulcers, pain, or swelling after surgery demonstrated statistically significant clinical efficacy. This phenomenon might be related to the mechanism of 5-ALA-PDT. In our study, five patients experienced significant pain and required analgesics, and only two patients showed notable local swelling and required oral prednisone. This suggests that the adverse reactions to the 5-ALA-PDT treatment are relatively mild, which is consistent with the findings of Yao et al.^25^ However, this study did not employ standardized instruments such as the Visual Analog Scale (VAS) or the Numeric Rating Scale (NRS) to assess pain, nor did it utilize patient-reported outcome measures like the Oral Health Impact Profile (OHIP-14) to evaluate quality of life. Pain assessment relied primarily on subjective complaints documented in medical records, which compromised precision; moreover, therapeutic efficacy for OL was not captured through quantitative values. These constitute limitations of the present study. Therefore, in future investigations, our research group should incorporate the aforementioned standardized assessment tools to enable a more accurate quantitative evaluation of pain, quality of life, and treatment outcomes.

Pietruska et al^17^ used Photofrin (containing 20% dihematoporphyrin e6) mediated PDT to treat 44 OL patients (with a power of 300 mW and a wavelength of 660 nm). Among these patients, 12 cases were completely cured, 22 cases were partially cured, and 10 cases showed no changes. Their study found that PDT was more effective for lesions located on the lips and cheeks compared to those on the tongue and gingiva. The current study analyzed the correlation between the location of the lesion and the effectiveness of 5-ALA-PDT treatment. A higher treatment effectiveness rate was found for lesions occurring on the ventral tongue. This result is opposite to that of Pietruska et al,^17^ which might be due to the different photosensitizers used or potentially to the smaller sample size in our study. Our study did not include any cases of lesions on the lips, which could have contributed to the different outcomes.

After treatment with 5-ALA-PDT, OL patients were followed up for 3 to 25 months. This wide range is inherent to the prospective enrollment design: patients were recruited over a defined period, and the study had a fixed closing date for data analysis. Consequently, those enrolled later had naturally shorter follow-up durations at the time of analysis, whereas those enrolled early had longer follow-up. Three cases of recurrence were observed, occurring at 1, 2, and 4 months’ post-treatment, resulting in a recurrence rate of 11.5%. This finding is consistent with the research of Yao et al,^25^ who found that the peak period for OL lesion recurrence after 5-ALA-PDT treatment was between 3-6 months post-operatively. This similarity suggests that patients should be revisited monthly within the first 6 months after treatment, and then every 3-6 months thereafter. Although 5-ALA-PDT treatment has shown good initial efficacy in treating OL, recurrence still occurs and its mechanism is not yet clear. Our study explored the correlation between OL recurrence and basic clinical characteristics, finding that the recurrence rate of moderate epithelial dysplasia was higher than that of simple hyperplasia and mild epithelial dysplasia, and that non-homogeneous lesions had a higher recurrence rate than homogeneous lesions (p<0.05). However, there was no statistically significant difference in short-term clinical effectiveness between these two, which might be due to the limited depth and range of action of 5-ALA-PDT. Although it can remove visible OL lesions, it might be less effective for deeper infiltrated OL tissues or cells in the long run.^2,10,12^ This suggests that for non-homogeneous OL lesions or those accompanied by abnormal proliferation, judging clinical efficacy solely based on changes in lesion size before and after treatment may overestimate the effectiveness of treatment. Therefore, further studies may be required, using non-invasive or minimally invasive auxiliary detection methods such as toluidine blue, VEL scope, and DNA quantitative analysis to comprehensively evaluate the treatment effectiveness of OL. This will be the focus of our team’s future research. Regarding the management of OL lesion recurrence, options include continued photodynamic therapy or surgical excision. For patients with initial recurrence, our study opted for continued photodynamic therapy, while for those with repeated recurrence, surgical excision was chosen.

In this study, four patients with partial remission or recurrence (including two with simple hyperplasia and two with moderate epithelial dysplasia) underwent a second biopsy. Among them, two patients (one with simple hyperplasia and one with moderate epithelial dysplasia) developed malignant transformation after 5-ALA-PDT. Case 1 was a 28-year-old male smoker. The initial biopsy revealed simple hyperplasia, and the lesion was located on the ventral tongue. After three sessions of 5-ALA-PDT, the lesion resolved but recurred one month postoperatively. Six months after a second course of treatment, no visible leukoplakia was observed; however, the patient reported discomfort at the original site during swallowing. A second biopsy indicated local malignant transformation. Case 2 was a 67-year-old male smoker with a histopathological diagnosis of moderate epithelial dysplasia, and the lesion was also located on the ventral tongue. The lesion resolved after three sessions of 5-ALA-PDT but recurred two months postoperatively. Six months after a second course of treatment, no obvious leukoplakia was visible, however, the patient experienced discomfort during swallowing and speaking. A second biopsy revealed local malignant transformation. Tovaru et al^21^ reported a malignant transformation rate of approximately 7.5% for oral leukoplakia and suggested that biopsy is warranted even for lesions that appear homogeneous and clinically benign. The malignant transformation rate in the present study was 6.5%; however, because repeat biopsy was performed only in selected cases presenting with recurrence or local discomfort, this rate may be underestimated. Shafirstein et al^20^ treated 17 OL lesions with a diameter ≥ 10 mm using PDT, and the immunohistochemical results showed that the expression of the tumor suppressor gene p53 increased in 73% of the tissue samples, and in 58% of the samples, the expression of the cell proliferation nuclear antigen Ki-67 decreased. This indicates that PDT can delay the carcinogenic process of OL. However, in the present study, the second biopsy of the four patients did not show any improvement in the degree of dysplasia compared to the first biopsy, and two patients developed malignant transformation. Wong et al^24^ treated 11 OL patients with PDT, and shortly after the surgery, 3 patients progressed to oral squamous cell carcinoma. Wong et al^24^ proposed that although 5-ALA-PDT can effectively remove potential lesions, it cannot effectively reduce the canceration rate of OL due to its regional canceration effect. The inconsistency of these conclusions^20,24^ may be attributed to the small sample size. Therefore, whether 5-ALA-PDT can reduce the malignancy conversion rate of OL requires long-term follow-up and further experimental validation. Because only two patients in this study underwent malignant transformation, statistical analysis of potential risk factors could not be performed. In the future, our research group will expand the sample size to further investigate risk factors associated with malignant transformation. Nevertheless, this study suggests that clinicians should closely monitor patients after 5-ALA-PDT treatment, especially those with lesions in “high-risk areas” such as the ventral tongue or those experiencing local pain.

This study demonstrated that 5-ALA-PDT is highly effective in treating OL, with advantages such as ease of operation, rapid onset, and minimal trauma. To further contextualize our findings within the broader landscape of OL management, we systematically compared our protocol with existing treatment modalities. In this study, the optimized 5-ALA-PDT protocol achieved an effective rate of 90.3% and a complete response rate of 61.3%, which were higher than the 86.2% and 55.2% reported by Han et al.^8^ Compared with the pooled recurrence rates after surgical excision (29.5%) and laser excision (32.2%) reported in the meta-analysis by Zhou et al,^27^ the recurrence rate of our protocol was lower (11.5%). The malignant transformation rate of 6.5% was close to the pooled rates for surgical excision (8.9%) and laser therapy (6.0%); however, given the limited sample size, this finding requires further confirmation. Regarding other laser ablation modalities, CO₂ laser and Er:YAG laser are widely used but are also associated with high recurrence rates and an inability to assess resection margins.^1^ Kawczyk et al^9^ compared photodynamic therapy with cryotherapy for OL and reported a complete response rate of 72.9% and a recurrence rate of 27.1% for the PDT group, compared with 89.2% and 24.3%, respectively, for the cryotherapy group. They concluded that both modalities are comparable and may serve as alternatives to conventional surgery, but PDT offers greater convenience, less pain, and better esthetic outcomes. The 5-ALA-PDT protocol used in this study demonstrated clear minimally invasive advantages; however, its limited penetration depth in non-homogeneous lesions may lead to overestimation of treatment efficacy. With respect to different photosensitizers, Pietruska et al^17^ treated 44 OL cases with Photofrin-mediated PDT containing 20% dihematoporphyrin e6 and achieved a complete response rate of 27.3%, which was lower than the 61.3% observed in our study, possibly because of differences in photosensitizer properties and light-source parameters. Currently, high-level evidence directly comparing 5-ALA-PDT with surgery or different laser therapies is lacking. In the future, our team intends to expand the sample size, establish control groups, extend the follow-up period, and further validate and supplement the findings regarding factors influencing the clinical efficacy of 5-ALA-PDT for OL, as well as its role in preventing lesion recurrence and malignant transformation, with the aim of providing therapeutic references and a theoretical basis for the clinical application of this therapy.

## CONCLUSIONS

5-ALA-PDT has shown appears to be an effective treatment in treating OL, achieving a response rate of 90.3% in this study. The therapeutic outcomes are correlated with lesion location and postoperative reactions. Patients with OL lesions <2 cm^2^ achieved clinical cure in shorter treatment courses compared to those with lesions ≥ 2 cm^2^. As a novel therapeutic approach, 5-ALA-PDT offers advantages including convenient operation, rapid onset, and minimal invasiveness, making it a promising treatment option for OL patients. Nevertheless, further research is required to evaluate its long-term efficacy in preventing lesion recurrence and malignant transformation.

## ACKNOWLEDGEMENTS

This work was supported by the National Natural Science Foundation of China (82260185) and the Guizhou Provincial Natural Science Foundation (ZK[2023]340).
